# Protocol for a multicentre, parallel-arm, 12-month, randomised, controlled trial of arthroscopic surgery versus conservative care for femoroacetabular impingement syndrome (FASHIoN)

**DOI:** 10.1136/bmjopen-2016-012453

**Published:** 2016-08-31

**Authors:** D R Griffin, E J Dickenson, P D H Wall, J L Donovan, N E Foster, C E Hutchinson, N Parsons, S Petrou, A Realpe, J Achten, F Achana, A Adams, M L Costa, J Griffin, R Hobson, J Smith

**Affiliations:** 1 University of Warwick, University Hospitals of Coventry and Warwickshire NHS Trust, Coventry, UK; 2 Warwick Medical School, University of Warwick, Coventry, UK; 3 University of Bristol, University Hospitals Bristol NHS Foundation Trust, Bristol, UK; 4 Arthritis Research UK Primary Care Centre, Research Institute of Primary Care and Health Sciences NIHR, Keele University, Keele, UK

**Keywords:** femoroacetabular impingement, hip impingement, hip arthroscopy, physiotherapy

## Abstract

**Introduction:**

Femoroacetabular impingement (FAI) syndrome is a recognised cause of young adult hip pain. There has been a large increase in the number of patients undergoing arthroscopic surgery for FAI; however, a recent Cochrane review highlighted that there are no randomised controlled trials (RCTs) evaluating treatment effectiveness. We aim to compare the clinical and cost-effectiveness of arthroscopic surgery versus best conservative care for patients with FAI syndrome.

**Methods:**

We will conduct a multicentre, pragmatic, assessor-blinded, two parallel arm, RCT comparing arthroscopic surgery to physiotherapy-led best conservative care. 24 hospitals treating NHS patients will recruit 344 patients over a 26-month recruitment period. Symptomatic adults with radiographic signs of FAI morphology who are considered suitable for arthroscopic surgery by their surgeon will be eligible. Patients will be excluded if they have radiographic evidence of osteoarthritis, previous significant hip pathology or previous shape changing surgery. Participants will be allocated in a ratio of 1:1 to receive arthroscopic surgery or conservative care. Recruitment will be monitored and supported by qualitative intervention to optimise informed consent and recruitment. The primary outcome will be pain and function assessed by the international hip outcome tool 33 (iHOT-33) measured 1-year following randomisation. Secondary outcomes include general health (short form 12), quality of life (EQ5D-5L) and patient satisfaction. The primary analysis will compare change in pain and function (iHOT-33) at 12 months between the treatment groups, on an intention-to-treat basis, presented as the mean difference between the trial groups with 95% CIs. The study is funded by the Health Technology Assessment Programme (13/103/02).

**Ethics and dissemination:**

Ethical approval is granted by the Edgbaston Research Ethics committee (14/WM/0124). The results will be disseminated through open access peer-reviewed publications, including *Health Technology Assessment*, and presented at relevant conferences.

**Trial registration number:**

ISRCTN64081839; Pre-results.

**Strengths and limitations of this study**

- This trial is multicentre, pragmatic and randomised, making results generalisable across the NHS.
- Further strengths include a large sample size and the robust procedures to assess treatment fidelity.
- The trial has a large sample size (344).
- There are robust procedures to assess treatment fidelity.

## Background

Until recently, there was little understanding of the causes of hip pain in young adults. Since first described in 2003, there has been increasing recognition of the syndrome of femoroacetabular impingement (FAI), which seems to account for a proportion of the previously undiagnosed cases of hip pain in young adults.^1^ ^2^ Subtle deformities of hip shape combine to cause premature contact between the femoral neck and the acetabular rim which may result in hip pain.^1^ ^3^ These shape abnormalities typically divide into three categories:^3^ ^4^

- Cam-type, in which the femoral head is oval rather than round, or there is bony prominence on the femoral neck;
- Pincer-type, in which the rim of the acetabulum is excessively prominent, in one or more areas of its circumference;
- Mixed-type hip impingement, a combination of cam and pincer types.

Surgery can be performed to reshape the bony contour of the proximal femur and/or acetabular rim in order to prevent impingement. Surgery for FAI has evolved more quickly than our understanding of the epidemiology or natural history of the condition,^5^ ^6^ and is becoming an established treatment for FAI.^7^ The risks of complications from open surgery are greater than those for arthroscopic surgery and current evidence suggests that the outcomes of arthroscopic treatment for the symptoms of FAI are comparable to open surgery.^8^ ^9^ Consequently, hip arthroscopy for FAI is a rapidly growing new cost pressure for health providers.^10^ However, a recently published Cochrane review highlighted the absence of randomised controlled trials (RCTs) comparing FAI surgery with conservative care such as physiotherapist-led exercise.^11^

Physiotherapy has also been shown to be beneficial in patients with FAI syndrome.^12^ ^13^ During a successful feasibility study (HTA 10/41/02), a programme of physiotherapist-led conservative care was developed called personalised hip therapy (PHT).^14^

### Aims of trial

We aim to compare the clinical and cost-effectiveness of arthroscopic surgery versus physiotherapist-led conservative care (PHT) in patients with symptoms of FAI syndrome.

## Methods/design

This trial will be conducted in accordance with the Medical Research Council's Good Clinical Practice principles and guidelines, the Declaration of Helsinki, Warwick Clinical Trials Unit (WCTU) standard operating procedures (SOPs), relevant UK legislation and the trial protocol. Ethical approval was granted on 1 May 2014 (14/WM/0124), by the Edgbaston Research Ethics committee (current approved protocol V.3.1 20/01/2016). The trial will be reported in line with the CONSORT statement. This full trial follows a successful feasibility and pilot trial (HTA10/41/02).^14^

### Trial design and setting

This is a protocol for the **f**ull UK RCT of **a**rthroscopic **s**urgery for **h**ip **i**mpingement versus best c**on**servative care (**FASHIoN**). We will conduct a multicentre, pragmatic, assessor-blinded, parallel arm, 12 months, 1:1 RCT of hip arthroscopy versus conservative care for FAI assessing patient pain, function, general health, quality of life, satisfaction and cost-effectiveness. There is an integrated qualitative recruitment intervention that includes interviews with recruiters and patients, and observations of recruitment appointments to ensure patients have the opportunity to fully consider participation in the trial.^15^

We hypothesise that arthroscopic surgery is superior to conservative care at 12 months for self-reported hip pain and function for patients with FAI syndrome. The trial will be conducted on consenting patients treated in the NHS. Hospitals participating in FASHIoN will have an organised hip arthroscopy service treating at least 20 patients with arthroscopic surgery for FAI per year.

### Target population

We intend to recruit a cohort of typical patients with FAI deemed suitable for arthroscopic surgery. This included patients who may have already received a course of physiotherapy.

#### Inclusion criteria

- Age ≥16 (no upper age limit);
- Symptoms of hip pain—patients may also have symptoms of clicking, catching or giving way;
- Radiographic evidence of pincer- and/or cam-type FAI morphology on plain radiographs and cross-sectional imaging, defined as:
  Cam morphology—an α angle >55°;^16^
  Pincer morphology—a lateral centre edge angle of >40° or a crossover sign on the anteroposterior radiograph of the pelvis;^17^
- The treating surgeon believes the patient would benefit from arthroscopic FAI surgery;
- The patient is able to give written informed consent and to participate fully in the interventions and follow-up procedures.

#### Exclusion criteria

- Evidence of pre-existing osteoarthritis, defined as Tonnis grade >1,^18^ or more than 2 mm loss of superior joint space width on anterio-posterior pelvic radiograph;^19^
- Previous significant hip pathology such as Perthes' disease, slipped upper femoral epiphysis or avascular necrosis;
- Previous hip injury such as acetabular fracture, hip dislocation or femoral neck fracture;
- Previous shape changing surgery (open or arthroscopic) in the hip being considered for treatment.

### Participant identification, invitation, recruitment and baseline data collection

Patients who complain of hip pain, who do not already have a diagnosis of hip osteoarthritis, will be identified as potential participants by screening referral letters to collaborating surgeons. Research nurses/associates will keep accurate screening logs to identify if these potential participants meet the eligibility criteria. Once diagnosed with FAI syndrome by the surgeon, and deemed eligible for the trial, the patient will be given a trial information sheet (see online supplementary files 1 and 2) and referred to a trained recruiter for a trial information consultation. During this consultation, patients can discuss the trial, participation will be offered and informed consent obtained (see online supplementary files 3 and 4). It will be explained that participation is voluntary and patients can withdraw at any time. Once consent is obtained, and prior to treatment allocation, baseline patient reported outcomes will be collected (see the Outcome measures section).

10.1136/bmjopen-2016-012453.supp1 Supplementary data

10.1136/bmjopen-2016-012453.supp2 Supplementary data

10.1136/bmjopen-2016-012453.supp3 Supplementary data

10.1136/bmjopen-2016-012453.supp4 Supplementary data

**Figure 1:**
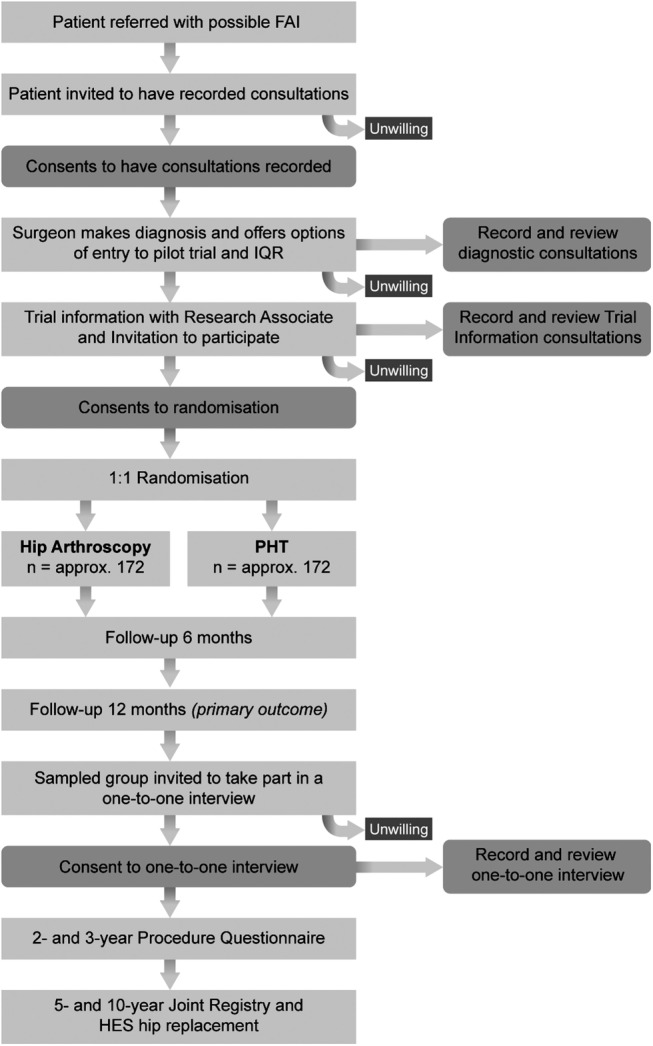
Participant flow diagram.

In order to optimise recruitment and informed consent, trained qualitative researchers will observe recordings of the surgeons' and research associate/nurses' trial information consultations (see online supplementary files 2 and 4), to identify communication patterns that facilitate or hinder patient recruitment^15^ (see figure 1). In-depth interview with the recruiters will be undertaken to identify clear obstacles and hidden challenges to recruitment, including the influence of patient preferences and equipoise.^20^ Research teams will be interviewed to identify clinician equipoise, patient pathway from eligibility to consent and staff training needs at each participating site.^15^ Findings will be fed back to the CI and trial management group, so that practice can be reviewed and any necessary changes (including additional training) implemented. The number of eligible patients, the percentages of these that are approached and consented to be randomised will be monitored at each site.

This research will be linked, through Donovan, to the Quintet programme of research within the MRC ConDuCT-II (Bristol) Trial Methodology Hub.

### Randomisation

Participants will be randomised, in a 1:1 ratio, to arthroscopic surgery or PHT using a computer-generated sequence. Allocation will be made by the research nurse/associate via a centralised telephone randomisation service provided remotely by WCTU. Allocation concealment will be ensured, as the randomisation programme will not release the randomisation code until the patient has been recruited into the trial. In order to improve baseline balance between intervention group samples, a minimisation (adaptive stratified sampling) algorithm will be implemented using study site and impingement type (cam, pincer or mixed) factors. Research nurses/associates who recruit participants will ensure they are referred for the allocated intervention. Patients and clinicians cannot be blind to treatment allocation. However, outcome assessors will be blind to the treatment delivered.

### Interventions

The two interventions will start as soon as possible after randomisation. We will record dates of randomisation and the start of allocated treatment. As this is a pragmatic trial, participants were not prohibited from undergoing any additional/concomitant care.

#### Arthroscopic surgery

Arthroscopic surgery will be completed by a Consultant Surgeon delivering hip arthroscopy as part of their routine practice. Arthroscopic hip surgery will be performed under general anaesthesia according to the surgeon's usual practice. Shape abnormalities and consequent labral and cartilage pathology will be treated. Bony resection at the acetabular rim and at the head–neck junction will be assessed by intraoperative image intensifier radiographs and/or satisfactory impingement-free range of movement of the hip. Patients will be allowed home when they can walk safely with crutches (usually within 24 hours). On discharge, patients will be referred for a course of rehabilitation as per usual care for that surgeon. We will not specify a protocol for this postoperative physiotherapy, but will record the surgeons' routine postop care and any case-by-case changes to this. Care will be taken to ensure that physiotherapists delivering postoperative care to FASHIoN trial participants are different from those trained and providing PHT in order to avoid contamination between groups. Patients will also have a postoperative MRI after 6 weeks.

In order to ensure the fidelity of the surgery and to identify participants for a secondary analysis, a panel of international experts will review operation notes, intraoperative images and postoperative MRI scans to assess whether adequate surgery was undertaken. This panel includes: Mark Philippon (USA), Martin Beck (Switzerland), John O'Donnell (Australia) and Professor CEH (UK).

#### Personalised hip therapy

PHT is a package of physiotherapist-led best conservative care for FAI. It was developed during the feasibility study and ‘road-tested’ during the pilot trial (HTA 10/41/02).^14^ The care being offered represents a consensus of what physiotherapists, physicians and surgeons regard as ‘best conservative care’ for FAI. PHT will be delivered by a senior physiotherapist at each site, who will be trained at a FASHIoN PHT workshop, and supported in PHT delivery by a physiotherapy research facilitator.

PHT consists of four key components:

1. An assessment of pain, function and range of hip motion,
2. Patient education and advice,
3. Help with pain relief (which may include up to one radiographic-guided intra-articular steroid injection where pain prevents performance of the exercise programme),
4. An exercise programme that has the key features of individualisation, progression and supervision.

The intervention is delivered over a minimum of six patient contacts (at least three of which must be face-to-face treatment contacts, others can be by telephone and email) over a period of 6 months. In situations where the patient needs additional review, support or guidance, further sessions with the physiotherapist are permitted up to a maximum of 10 contacts. Evidence of exercise individualisation, supervision and progression will be sought from individual participant physiotherapy case report forms (CRFs). Accuracy of CRFs will be audited against the physiotherapist's treatment notes.

The PHT CRFs will be assessed for intervention fidelity to identify participants for a secondary analysis by the panel that developed the protocol for PHT, including: Professor NEF (Senior Academic Research Physiotherapist), Ivor Hughes and David Robinson (UK; Extended Scope Musculoskeletal Physiotherapists) and PW (Academic Orthopaedic Surgeon).

Crossover of participants between interventions can be problematic in trials of this nature. In order to minimise, this care will be taken prior to enrolment in the trial to ensure potential participants:

- Are willing to receive either intervention,
- Understand both treatments are thought to provide benefit,
- Are willing to remain with their allocation for 12 months,
- Understand that both interventions may take 6 months to improve symptoms.^12^ ^19^

In instances where patients are not satisfied with how their treatment is progressing prior to reaching the primary outcome, they will be able to have a further consultation with their treating surgeon where they would be treated in their best interests.

### Risks and benefits

Both interventions are thought to provide benefit in patients with FAI. The short-term risks of this study relate to the two interventions. These risks are described below and inform the expected serious adverse events (SAEs).

Hip arthroscopy requires a general anaesthetic. The risk of complications from hip arthroscopy is about 1–2%. These include:

- Infection—thought to be <1 in 1000.
- Bleeding—possibly causing bruising or a local haematoma.
- Traction-related—in order to perform hip arthroscopy, traction is required to separate the hip joint surfaces. Sometimes after the procedure, the pressure from the traction can cause some numbness in the leg. The numbness usually resolves within a few hours or days.
- Osteonecrosis—during surgery, the blood supply to the hip joint could be damaged. However, there are no reported cases of osteonecrosis following arthroscopic FAI surgery.
- Femoral neck fractures—this is also a very rare complication. This complication would require a further procedure to fix the fracture.

#### Personalised hip therapy

There are some small risks with pain medications and joint injection. However, the main risk is muscle soreness and transient increases in pain from the exercises that will be undertaken.

### Outcome measures

Baseline data will be collected from participants once consent is obtained and prior to randomisation. Follow-up questionnaires will be administered centrally by a data clerk via post. If participants fail to respond, they will be contacted via telephone, email or via their next of kin where necessary. Table 1 lists the data collected and at which follow-up time points.

**Table 1:** Data collection time points

Time point	Data collection
Baseline	Demographics, physical activity (UCLA Activity Scale),^29^ iHOT-33, SF-12,EQ-5D Preoperative imaging, economics questionnaire
Intervention	Operation notes and photographs; or PHT log. Complications records 6 weeks post start of intervention. Postoperative MRI (surgery intervention only)
6 months	iHOT-33, SF-12, EQ-5D, resource usage, adverse events
12 months (primary outcome)	iHOT-33, SF-12, EQ-5D, patient satisfaction, resource usage, adverse events
2 years	Further procedures questionnaire
3 years	Further procedures questionnaire
5 and 10 years	Linkage to National Joint Registry and HES to identify need for hip replacement

### Primary outcome

The primary outcome measure is hip pain, function and hip-related quality of life using the **International Hip Outcome Tool-33** (iHOT-33) at 12 months following randomisation. iHOT-33 is a validated hip-specific patient-reported outcome tool which measures health-related quality of life in young, active patients with hip disorders.^21^ It consists of the following domains: symptoms and functional limitations, sports and recreational activities, job-related concerns and social, emotional and lifestyle concerns.

We chose it following our feasibility and pilot study as:

- It is more sensitive to change than other hip outcome tools,^21^
- It does not show evidence of floor or ceiling effects in patients undergoing hip arthroscopy,^21^
- Patients were involved extensively in item generation; so we can be confident that it measures what is most important to patients,^21^
- There is an independently determined minimally clinically important difference (MCID),^21^
- It is used as the principal outcome measure for the UK Non-Arthritic Hip Registry; mandated for arthroscopic FAI surgery by the National Institute of Health and Care Excellence (NICE).^10^

### Secondary outcome measures

#### Health-related quality of life: EQ-5D 5L

This is a validated measure of health-related quality of life, consisting of a five-dimensional health status classification system and a separate visual analogue scale. EQ-5D is applicable to a wide range of health conditions and treatments and provides a simple descriptive profile and a single index value for health status.^22^ Responses will be converted into health utility scores using established algorithms.^23^

#### *General health*: Short Form-12 Health Survey V.2

This is a validated and widely used health-related quality-of-life measure, particularly including hip conditions and treatments.^24^ SF-12 is able to produce the physical and mental component scales originally developed from the SF-36 with considerable accuracy but with far less respondent burden.^25^ Responses will be converted into health utility scores using established algorithms.^26^

#### Patient satisfaction

Using questions that our team (Foster) has used in previous trials with musculoskeletal pain patients,^27^ we will measure two distinct dimensions of satisfaction in all participants during follow-up: ‘*Overall, how satisfied are you with the treatment you received?’* and ‘*Overall*, *how satisfied are you with the results of your treatment?’* Responses are on a 5-point Likert scale. These questions are in line with previous studies of patient satisfaction which show that the majority of patients express overall satisfaction with the care they received, but fewer express overall satisfaction with the clinical outcomes resulting from their care.

#### Qualitative assessment of outcome

We will conduct in-depth interviews one-to-one with a purposively selected sample of 25–30 participants in each of the trial groups, including older and younger, male and female, more and less active and more and less satisfied participants recruited at different trial sites. The qualitative interviews will supplement the quantitative outcomes. Interviews will explore experiences of the trial processes, the treatments and the consequences of treatment to participants' lives, health and well-being.

#### Adverse events

We will record the number and type of adverse events (AEs) up to 12 months. Any AEs will be reported on the appropriate CRF and returned to WCTU. Any SAEs will be faxed to WCTU, within 24 hours of the local investigator becoming aware, where the Chief Investigator will determine causality and expectedness. SAEs deemed unexpected and related to the trial will be reported to the research ethics committee within 15 days.

#### Resource usage

Information on healthcare resource use will be collected by incorporating questions within the patient follow-up questionnaires. We confirmed the feasibility and acceptability of this approach in our pilot trial, and patient self-reported information on service use has been shown to be accurate in terms of the intensity of use of different services.^28^

#### Need for further procedures

We will record any further treatments performed in both groups, such as hip arthroscopy, open hip preservation surgery, hip replacement or additional ‘out of trial’ physiotherapy. We propose to ascertain the need for further procedures by questionnaire at 2 and 3 years. We also propose a 5 and 10-year no-cost ascertainment of hip replacement by linkage to the UK National Joint Registry (NJR) and Hospital Episode Statistic (HES) databases.

### Sample size calculation

The development work for iHOT-33 reported a mean iHOT-33 score of 66 and an SD of 19.3 in a heterogeneous population with a variety of hip pathologies. The baseline iHOT-33 data from our pilot trial (HTA grant 10/41/02) suggests the target population of patients being considered for hip arthroscopy for FAI have lower scores with less variability, with a mean of 33 and SD of 16. The MCID for iHOT-33 in this population is 6.1 points.^21^

Our sample size calculation is therefore based on an SD of 16 and a between-group MCID of 6.1: a standardised effect difference between groups at 12 months of 0.38. The expected sample size for 90% power to detect an effect size of 0.38 at 12 months, at a 5% significance level, assuming an approximately normal distribution of the iHOT-33 score is 292. Allowing for 15% loss to follow-up at 12 months, we will recruit a sample of 344 participants over 26months in the UK (172 in each group).

### Statistical analysis

The primary analysis will be of differences in hip-related quality of life (iHOT-33) at 12 months between the two treatment groups, blinded, on an intention-to-treat basis and presented as the mean difference between the trial groups with a 95% CI. iHOT-33 data will be assumed to be normally distributed; possibly after appropriate variance-stabilising transformation.

The minimisation randomisation procedure should ensure treatment group balance across recruiting sites. We have no reason to expect that clustering effects will be important for this study, but the possibility of such effects will be explored as part of the analysis.^33^ We plan to account for clustering by generalising a conventional linear (fixed-effects) regression approach to a mixed-effects modelling approach; where patients are naturally grouped by recruiting sites (random-effects) and, if amenable to analysis, also by physiotherapist and surgeon. This model will formally incorporate terms that allow for possible heterogeneity in responses for patients due to the recruiting centre, in addition to the fixed effects of the treatment groups, and patient characteristics that may prove to be important moderators of treatment effect such as age, gender and FAI type. This analysis will be conducted using specialist mixed-effects modelling functions available in the software packages Stata (StataCorp. 2015. Stata Statistical Software: Release 14. College Station, Texas, USA: StataCorp LP) and R (http://www.r-project.org/). All tests will be two-sided and considered to provide evidence for a statistically significant difference if p values are <0.05 (5% significance level).

Secondary analyses will be performed using the above strategy for other approximately normally distributed outcome measures, including iHOT-33 at 6 months, SF-12 (and computed subscales) and EQ5D. Differences in dichotomous outcome variables such as AEs, complications related to the trial interventions and the need for further procedures will compared between groups using χ^2^ tests (or Fisher's exact test) and mixed-effects logistic regression analysis will be undertaken, adjusting for the stratifying variables, with differences between trial intervention groups quantified as ORs (and 95% CIs). The temporal patterns of any AEs will be presented graphically and if appropriate, a time-to-event analysis (Kaplan-Meier survival analysis) will be used to assess the overall risk and risk within individual classes of AEs. Ordinal scores for patient satisfaction will be compared between intervention groups using proportional odds logistic regression analysis, assuming that the estimated intervention effect between any pair of categories is equivalent.

Our inferences will be drawn from the intention-to-treat analysis. We will perform two exploratory secondary analyses. One will compare patients who received surgery and those who received conservative care. A second exploratory analysis will compare patients randomised to surgery or PHT and received treatment deemed to be of a high fidelity by the respective review panels. We plan to perform a subgroup analysis by FAI type because it is possible that treatment effect is moderated by type. We anticipate that adequate steps have been taken to prevent crossovers from being a major issue for this study. Therefore, we expect the main intention-to-treat analysis to provide definitive results. An independent Data Monitoring Committee (DMC) will monitor crossovers and adherence to treatment and advise on appropriate modifications to the statistical analysis plan as the full trial progresses.

The initial feasibility and pilot studies (HTA 10/41/02) were designed explicitly to assess feasibility and measure recruitment rates, and not to estimate treatment effectiveness. Data from the pilot will be pooled with data from the full trial, and analysed together.

### Economic analysis

An economic evaluation will be integrated into the trial design and will be conducted from the recommended NHS and personal social services perspective.^30^ Cost-effectiveness will be calculated using both within trial and lifetime horizons. Data will be collected on the health and social service resources used in the treatment of each trial participant until 12 months.

An incremental cost-effectiveness analysis, expressed in terms of incremental cost per quality-adjusted life year gained, will be performed. Results will be presented using incremental cost-effectiveness ratios and cost-effectiveness acceptability curves generated via non-parametric bootstrapping.

### Qualitative interview analysis

Participant interview transcripts will be analysed thematically, using methods of constant comparison derived from grounded theory.^31^ Emerging themes will be explored, looking for shared or disparate views among patients about their experiences, and among clinicians about their experiences of delivering the trial interventions. Focused conversation analysis will be undertaken on sections of recruitment appointments, and compared with the six-step good recruitment model developed in the pilot study to identify aspects of RCT presentation that are unclear, disrupted or hinder recruitment.^15^ ^20^ ^32^

### Data management

All of the data collected in this trial will be entered into a secure trial database held at WCTU. All data collected will be anonymised after the collection of baseline demographic data, and all participants given a unique trial number. Identifiable participant data will be held in a locked filing cabinet and coded with a trial participant number to tag identifiable data to the outcome data. The WCTU quality assurance manager will undertake audits of trial records in accordance with WCTU SOPs.

A DMC will be established comprised of members who are independent of the sponsor and who do not have competing interests. The DMC will review trial progress, interim data and safety aspects of the trial. They will also review the statistical analysis plan. Any recommendations will be fed back to the trial steering committee (TSC) by the DMC chair. Outcomes will not be analysed until all primary outcome data are collected. The trial may be stopped prematurely if mandated by the research ethics committee, the DMC or if funding ceases.

## Discussion

This protocol paper describes the FASHIoN trial; a multicentre RCT comparing hip arthroscopy to best conservative care (PHT) in order to establish the most clinically and cost-effective treatment for patients with FAI syndrome. Further details of the trial protocol can be found on the ISRCTN registry (ISRCTN64081839). This protocol will also be used for a randomised trial in Australia (ACTRN12615001177549). The results of the trial will be disseminated at international meetings and in peer-reviewed journals; to participants via post and to the public via the trial website.

The main strengths of this trial are that it is multicentre, pragmatic and randomised, making results generalisable across the NHS. Further strengths include a large sample size and the robust procedures to assess treatment fidelity.
