# Supplementary material for: Protocol for a multicentre, parallel-arm, 12-month, randomised, controlled trial of arthroscopic surgery versus conservative care for femoroacetabular impingement syndrome (FASHIoN)
Source: BMJ Open. 2016 Aug 31;6(8):e012453. doi: 10.1136/bmjopen-2016-012453 (PMC5013508; doi:10.1136/bmjopen-2016-012453)

<<<TO BE PRINTED ON LOCAL HEADED PAPER>>>

UK FASHIoN Study

Chief Investigator: Professor Damian Griffin

<u>Patient Information Sheet</u>

You are invited to take part in our research study. Before you decide whether to take part we would like you to understand why the research is being done and what it would involve for you. Once you have had a chance to read and absorb this information sheet a member of our team will personally go through the information with you and answer any questions you may have.

Background Information

Your hip joint has two bones that fit together like a ball in a socket, see figure 1.

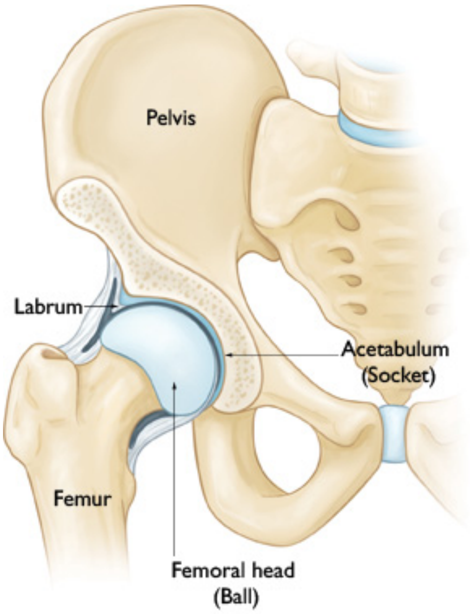

- Figure 1 – Normal Hip Joint

The bones that make the ball and socket joint are not the same shape in everyone. In some people with a shape similar to yours the bones press against each other and damage the local soft tissues such as the labrum (a soft cushioning around the hip joint- see figure 1) which can cause pain.

This is called Hip Impingement and the medical term for this is femoroacetabular impingement (FAI for short). Hip impingement has only been discovered in the last 10 years and we do not understand everything about the condition. Most importantly it is not clear what the best

treatment for hip impingement is. There are currently two treatment options available as standard care, physiotherapy and hip arthroscopy (explained below) and good results have been shown for both treatments, but we do not yet know if one is better than the other. There is thought to be a long-term risk of osteoarthritis in patients with hip impingement. It is not known if either of these two treatments (physiotherapy or hip arthroscopy) has any effect on this risk. In order to decide which treatment is better for patients like you in the future we need a study to compare these two treatments.

What is the purpose of this study?

This study aims to compare two different treatments for your condition - hip impingement:

- ***Personalised Hip Therapy*** – this is a new individualised and structured programme of exercise therapy designed for you by a physiotherapist. A more detailed description is provided later.
- ***Hip Arthroscopy*** – this is keyhole surgery and is designed to reshape the bone around your hip joint. A more detailed description is provided later

Why have I been invited to take part in the study?

We have invited 344 patients like you with hip impingement to take part in the study.

Do I have to take part?

It is up to you to decide whether or not to take part. If you do take part you can withdraw at any time and this will not affect the care you receive.

What will happen to me if I take part?

If you decide to take part you will be asked to sign a consent form. You will then be allocated to one of the two treatments. In order to make our study work it is crucial that we have equal numbers of volunteers in each treatment group and that the one you (are invited to) join is determined by a sophisticated machine designed for this purpose, and not influenced by us. More information about the two possible treatments is given below. Whichever treatment you have, please be assured that your care will be based on meeting your individual needs, and you will continue with the same team of physiotherapists and surgeons throughout. Both these teams work closely together and they will be able to monitor your progress and share information with one another about your individual case continually. During the study we will ask you to complete 3 short questionnaires by post. You will do one questionnaire before you begin treatment and then one at 6 and 12 months after your study entry. If you need help completing a questionnaire, a researcher can contact you by phone soon after you receive it to help you complete it. We will continue to monitor your progress after 12 months via two short questionnaires at 2 and 3 years. In addition, if you have hip arthroscopy as part of the study, we will arrange for you to have a further MRI scan of your hip after the surgery. The scan will happen at least 6 weeks after your surgery and will help us to analyse the surgery that has been undertaken

Which treatments are you comparing?

The two treatments that are being compared are:

- ***Personalised Hip Therapy***-This is a personalised programme of hip therapy that is supervised by a senior physiotherapist and designed to meet your individual needs. You may already have had a course of physiotherapy for your hip, however this programme of care is different and has been designed specifically to relieve pain in your hip and improve how it works. You will meet a senior physiotherapist with a specialist interest in hip impingement who will undertake a thorough assessment of your condition including the effect it has on your life. They will then customise a specific programme of hip exercises designed to help your hip. They will teach you these exercises in clinic and you will then be able to practise these exercises at home. This programme of exercises will gradually increase in intensity and difficulty so that by the end of the programme (12 weeks) we hope you will have developed improved control and strength around your hip with less pain. In addition to the hip exercise programme, a range of additional treatments will be offered to you. These include:

    - Techniques to improve the control and strength of your posture and walking
    - Personalised advice on techniques to modify the way you undertake daily activities
    - Specific advice about pain medications to help control your pain in the initial stages of the therapy, including the possibility of a steroid injection into the hip joint if required.

  The programme lasts 12 weeks and you will need to be able to attend the physiotherapy clinic at least 3 times to be assessed, and to have your treatment progressed by your physiotherapist. In addition to this, your physiotherapist will keep a close eye on your progress over the telephone and will contact you at least 3 times in order to ensure you progress well with the programme. The exercises you will be taught will focus on muscle control and balance in the first few weeks. You will then progress to resistance and stretching exercises and activity/sport-specific exercises in later stages of the programme. You and your physiotherapist will be able to arrange an additional 2 "booster" sessions of assessment / treatment if either of you feel that more time is required to undergo the therapy after the 12 week plan is over. Your physiotherapist may feel it necessary that you have an injection of local anaesthetic and steroid into the hip joint to provide additional pain relief to allow you to complete your treatment of personalised hip therapy. This would be conducted under either local anaesthetic in the skin or sedation provided by an anaesthetist.

Hip arthroscopy The procedure is done under a general anaesthetic (you will be put to sleep). The surgeon opens up a small passage through to your hip joint using special instruments introduced through incisions on the surface of your skin. A telescope is passed through these small passages, to look inside the hip, and further instruments are inserted that allow the surgeon to reshape the hip joint and repair locally damaged tissues, such as the labrum. You will normally need to stay in hospital for between 1-3 days after the procedure. Depending on the extent of surgery, some patients have to use crutches to walk for between 6-8 weeks after the procedure. There is a period

of rehabilitation after the procedure, which will be supervised by a physiotherapist in clinic, and practised at home. It will take between 2-3 months to complete the rehabilitation programme. In addition, we will arrange for you to have a further MRI scan of your hip after the surgery. The scan will happen at least 6 weeks after your operation and will help us to analyse the surgery that has been undertaken.

What are the possible risks of taking part?

The treatments are designed to help you, however, this cannot be guaranteed. The individual risks of each treatment are outlined below:

- **Personalised Hip Therapy -** There are some small risks with pain medications and joint injections. A hip joint injection carries a very small risk of infection and bruising. However, the main risk is muscle soreness and short-term increases in pain from the exercises that you will undertake. Generally the risks of this treatment are much lower than hip arthroscopy (surgery)

- **Hip Arthroscopy –** about 1 in 50 people have specific complications from hip arthroscopy. One very rare but serious risk is a break (fracture) of the hip during the surgery. If this happened you would need an additional operation to fix the break. Other risks of hip arthroscopy include:

  - Infection within the joint or around the wounds. This can sometimes be treated with antibiotics alone. In more serious cases it requires a further procedure to washout the hip.
  - Bleeding from the wounds, but this is usually a very small amount and quickly settles.
  - Numbness in groin, leg or foot. To undertake hip arthroscopy we need to apply a pulling force on your leg in order to access the hip joint. This can cause some numbness in your groin, leg or foot as a result. This usually resolves within a few hours or days after the procedure.

For this study, both treatment options may include the use of ionising radiation, but they are not in addition to what would normally be received if they were occurring outside of this study. Although all radiation you receive builds up over your lifetime, the small doses received from either of these treatments should not create a significant risk to your health. The maximum amount of radiation from either treatment is comparable to 40 normal chest X-Ray and equivalent to 3.5 months of exposure to natural background radiation.

How do these treatments work?

Personalised Hip Therapy – this therapy works by allowing soft tissues which are damaged and painful as a result of hip impingement, such as the labrum, a period of relative rest, so that they can heal naturally. This can take up to several weeks or months. During this period you will have learnt and practised many exercises that improve the movement and control of the hip and local joints (such as your lower back and pelvis), which should ensure that your hip impingement can no longer

occur, and that damaged soft tissues, such as the labrum, can continue to heal.Hip Arthroscopy – this procedure relies on surgically removing bits of bone from around the hip so that they no longer rub together and damage the soft tissues such as the labrum. Once the bits of bone have been removed, a period of rehabilitation is required so that the soft tissues can continue to heal.

One of the long-term concerns with hip impingement is that you have an increased risk of developing arthritis of the hip. It is really important that you know that at the moment we have no evidence that any treatment (including personalised hip therapy or hip arthroscopy) will have any effect on whether you subsequently develop arthritis of your hip. However by taking part in this study it will help us in the long term to determine if either of these two treatments can help prevent arthritis.

What if new information becomes available?

Sometimes during the course of a study, new information becomes available about the treatments that are being studied. If this happens, someone from our research team will tell you about it and discuss with you whether you want to continue in the study. If you decide to withdraw, you can discuss your continued care with your doctor. If you decide to continue in the study you might be asked to sign an updated consent form. Also, on receiving new information, we might consider it to be in your best interests to withdraw you from the study. If this happens we will explain the reasons to you and arrange for your care to continue.

What happens when the research study stops?

You will be in the study for one year. If you are still having problems after this time, we will arrange for you to see your hip specialist to continue your care.

What if something goes wrong?

In the event that something goes wrong and you are harmed during the research due to someone's negligence, then you may have grounds for legal action for compensation against the University of Warwick (contact Miss Nicola Owen, Deputy Registrar, 02476 522713) but you may have to pay your legal costs. The normal National Health Service complaints mechanisms will still be available to you. For independent advice contact the PALS service (Patient Advice Liaison Service) at Freephone 0800 0284203.

Will my taking part in this study be kept confidential?

All information which is collected about you during the course of the research will be kept strictly confidential, and will not be shared with anyone outside of your direct care team. Research data including your name and address will be sent to the University of Warwick so that research staff can stay in touch with you over the course of the year, and send you follow-up questionnaires at 6 and 12 months by post. These details will be sent from the hospital by secure means, and kept in locked filing cabinets or in password-protected computer databases accessible only to essential research

personnel at the University of Warwick. All other information about you which leaves the hospital will have your name and address removed so that you cannot be recognised from it. If you have hip arthroscopy an anonymised copy of your MRI will be sent electronically via a secure system to Clinical Graphics B.V. based in the Netherlands. If you agree, your GP and other doctors who may treat you, but are not part of this study, will be notified that you are taking part in this study.

What will happen to the results of the research study?

At the end of the study we will publish the findings in medical journals and at medical conferences. You will not be identified in any reports or publications resulting from the study. If you would like to obtain a copy of the published results, please contact the study coordinator Rachel Hobson on 02476-968629 or email: ukfashion@warwick.ac.uk

Who has reviewed this project?

This study has been reviewed and approved by NRES Committee West Midlands - Edgbaston. Approval was granted on 1st May, 2014.

Contacts for further information

If at any time, you would like further information about the study, you may contact the study coordinator, Rachel Hobson, by either telephoning 02476 968629 or emailing ukfashion@warwick.ac.uk

Or you can contact your local research lead, Mr Malviya Telephone 01670 529781 or Christine Dobb 0344 811 8111 extension 4561 or Professor Damian Griffin, who is the overall lead for this study on 02476 968618.

Where can I get additional information?

As well as the researcher and your surgeon who can provide advice and guidance, we have developed a website
http://www2.warwick.ac.uk/fac/med/research/csri/orthopaedics/research/fulllist/fashion/

This website provides additional useful information about hip impingement and its treatments, including a series of answers to frequently asked questions. In addition, it provides internet links to other trusted sources of information.

STEP by STEP guide to the study

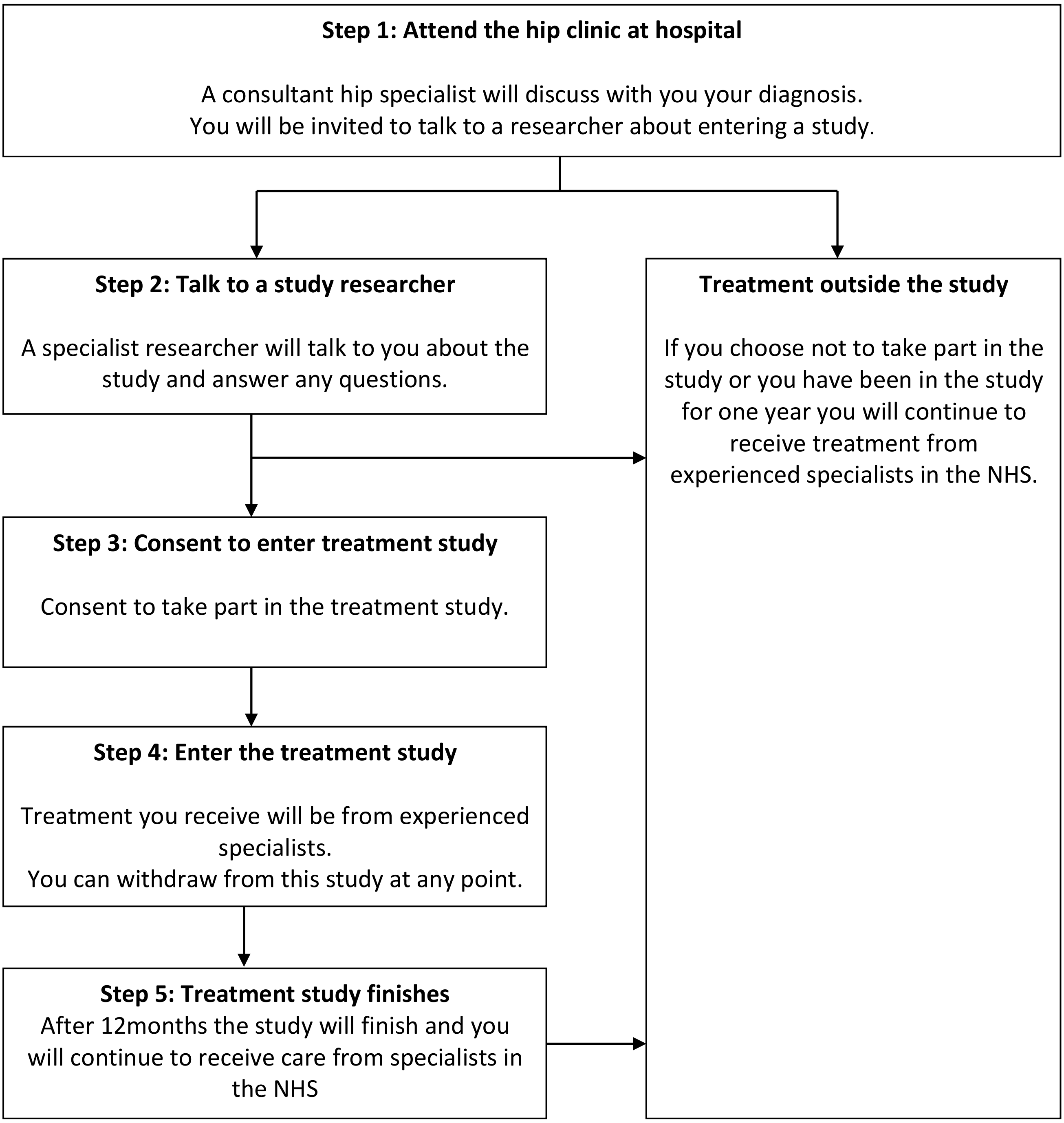

Supplement: Supplementary data [file bmjopen-2016-012453supp1.pdf]
