# Supplementary material for: Protocol for a multicentre, parallel-arm, 12-month, randomised, controlled trial of arthroscopic surgery versus conservative care for femoroacetabular impingement syndrome (FASHIoN)
Source: BMJ Open. 2016 Aug 31;6(8):e012453. doi: 10.1136/bmjopen-2016-012453 (PMC5013508; doi:10.1136/bmjopen-2016-012453)
Supplement: Supplementary data [file bmjopen-2016-012453supp2.pdf]

UK FASHIoN

Recording your Consultations

Chief Investigator Professor Damian Griffin

Patient Information Sheet

You are invited to take part in a research study. Your participation in the research is completely voluntary, but your involvement would help us to care for patients like you in the future. Before you decide whether to take part, it is important for you to understand why the research is being done and what it will involve – this is explained below.

What is the purpose of the research study?

The aim of this research study is to find out what information is explained to you by specialists in hip problems. The information we obtain, will help us plan future research studies looking at hip problems.

What will happen if I take part?

If you agree to take part, you will be asked to sign a consent form. During your consultations, your conversations will be recorded on a tape recorder. The recorded discussions will be written out and analysed by researchers at the University of Warwick. At any stage during the consultations, you may ask for the recording to be stopped without giving a reason.

What are the possible benefits to you of taking part?

There are no specific benefits for you in taking part. The information we get from this study will help us to plan future research studies in patients with hip problems.

Will my taking part remain confidential?

All information which is collected will be kept strictly confidential, it will not be shared with anyone outside of your direct care team. Copies of the anonymised interviews will be kept in a secure place, for 5 years, and then destroyed.

Do I have to take part?

It is up to you to decide whether or not to take part in the study. If you decide not to take part, this will not affect the standard of care you receive.

What if something goes wrong?

If you wish to complain, or have any concerns about any aspect of the way you have been approached or treated during the course of this study, you may contact Mrs Nicola Owen, deputy registrar at the University of Warwick on 02476 522785.

Who has reviewed this survey?

This study has been reviewed and approved by NRES Committee West Midlands -Edgbaston. Approval for this study was gained on 1st May, 2014.

Contacts for further information;

If you have any questions, please do not hesitate to ask your consultant at the start of your consultations. Or, for further information about this research project you may contact the study coordinator, Mrs Rachel Hobson by either telephoning 02476 968629 or emailing fashion@warwick.ac.uk, or Professor Damian Griffin, who is the overall lead of this study on 0247 6869618.

For independent advice contact the PALS service (Patient Advice Liaison Service) at freephone 0800 0284203.
