# Supplementary material for: Protocol for a multicentre, parallel-arm, 12-month, randomised, controlled trial of arthroscopic surgery versus conservative care for femoroacetabular impingement syndrome (FASHIoN)
Source: BMJ Open. 2016 Aug 31;6(8):e012453. doi: 10.1136/bmjopen-2016-012453 (PMC5013508; doi:10.1136/bmjopen-2016-012453)
Supplement: Supplementary data [file bmjopen-2016-012453supp3.pdf]

<<To be printed on local headed paper>>

UK FASHIoN

Chief Investigator: Professor Damian Griffin

CONSENT FORM – UK FASHIoN Study

Site ID [|] Participant ID: [| |]

Please **Initial** Box

1. I confirm that I have read and understand the information sheet dated 20th June, 2014– version 3 for the above study. I have had the opportunity to consider the information, ask questions and have had these answered satisfactorily. [ ]

2. I understand that my participation is voluntary and that I am free to withdraw at any time, without giving any reason, without my medical care or legal rights being affected. [ ]

3. I understand that relevant sections of any of my medical notes and data collected during the study may be looked at by responsible individuals from the University of Warwick, from regulatory authorities, or from the NHS trust, where it is relevant to my taking part in this research. I give permission for these individuals to have access to my records. [ ]

4. I understand that appropriate personal identifying information will be collected, stored and used by the study office to enable follow-up of my health status. This is on the understanding that any information will be treated with the strictest security and confidentiality. [ ]

5. I understand that information held and managed by The Health and Social Care Information Centre and other central UK NHS bodies may be used in order to help contact me or provide information about my health status. [ ]

6. I agree that encrypted anonymised copies of post-operative scans can be sent via electronic transfer to Clinical Graphics B.V. based in the Netherlands. [ ]

7. I agree to my GP being informed of my participation. [ ]

8. I agree to take part in the above study [ ]

___________________________	_____________	______________
Name of Patient	Date	Signature
___________________________	______________	______________
Name of Person taking consent	Date	Signature

Please ensure the following: -
Original consent form retained in the site file**, 1 copy** for Patient, **1 copy** for Hospital Notes
