# Supplementary material for: Protocol for a multicentre, parallel-arm, 12-month, randomised, controlled trial of arthroscopic surgery versus conservative care for femoroacetabular impingement syndrome (FASHIoN)
Source: BMJ Open. 2016 Aug 31;6(8):e012453. doi: 10.1136/bmjopen-2016-012453 (PMC5013508; doi:10.1136/bmjopen-2016-012453)
Supplement: Supplementary data [file bmjopen-2016-012453supp4.pdf]

<< To be printed on local headed paper>>

UK FASHIoN

Chief Investigator: Professor Damian Griffin

CONSENT FORM – Recording Your Consultation

Site ID	☐☐	Screening No.:	☐☐☐☐

Please **Initial** Box

1. I confirm that I have read and understand the information sheet dated 20th June, 2014 – version 3 for the above study. I have had the opportunity to consider the information, ask questions and have had these answered satisfactorily. ☐

2. I understand that my participation is voluntary and that I am free to withdraw at any time, without giving any reason, without my medical care or legal rights being affected. ☐

3. I give permission that anonymous quotes from my interview may be used in the reporting of this study. ☐

4. I give permission for the interview to be digitally-recorded. ☐

5. I agree to take part in the above study. ☐

____________________	____________	____________
Name of Patient	Date	Signature
____________________	____________	____________
Name of Person taking consent	Date	Signature

Please ensure the following:
Original consent form retained in the site file, 1 copy for Patient, 1 copy for Hospital Notes.
